# Detection of Tropical Diseases Caused by Mosquitoes Using CRISPR-Based Biosensors

**DOI:** 10.3390/tropicalmed7100309

**Published:** 2022-10-17

**Authors:** Salma Nur Zakiyyah, Abdullahi Umar Ibrahim, Manal Salah Babiker, Shabarni Gaffar, Mehmet Ozsoz, Muhammad Ihda H. L. Zein, Yeni Wahyuni Hartati

**Affiliations:** 1 Department of Chemistry, Faculty of Mathematics and Natural Sciences, Universitas Padjadjaran, Sumedang 45363, Indonesia; 2 Department of Biomedical Engineering, Near East University, Mersin 10, 99010 Nicosia, Turkey; 3 Department of Medical Biology and Genetics, Near East University, Mersin 10, 99010 Nicosia, Turkey

**Keywords:** CRISPR, electrochemistry, biosensor, tropical disease

## Abstract

Tropical diseases (TDs) are among the leading cause of mortality and fatality globally. The emergence and reemergence of TDs continue to challenge healthcare system. Several tropical diseases such as yellow fever, tuberculosis, cholera, Ebola, HIV, rotavirus, dengue, and malaria outbreaks have led to endemics and epidemics around the world, resulting in millions of deaths. The increase in climate change, migration and urbanization, overcrowding, and other factors continue to increase the spread of TDs. More cases of TDs are recorded as a result of substandard health care systems and lack of access to clean water and food. Early diagnosis of these diseases is crucial for treatment and control. Despite the advancement and development of numerous diagnosis assays, the healthcare system is still hindered by many challenges which include low sensitivity, specificity, the need of trained pathologists, the use of chemicals and a lack of point of care (POC) diagnostic. In order to address these issues, scientists have adopted the use of CRISPR/Cas systems which are gene editing technologies that mimic bacterial immune pathways. Recent advances in CRISPR-based biotechnology have significantly expanded the development of biomolecular sensors for diagnosing diseases and understanding cellular signaling pathways. The CRISPR/Cas strategy plays an excellent role in the field of biosensors. The latest developments are evolving with the specific use of CRISPR, which aims for a fast and accurate sensor system. Thus, the aim of this review is to provide concise knowledge on TDs associated with mosquitoes in terms of pathology and epidemiology as well as background knowledge on CRISPR in prokaryotes and eukaryotes. Moreover, the study overviews the application of the CRISPR/Cas system for detection of TDs associated with mosquitoes.

## 1. Introduction

The world is constantly facing the outbreak and reemergence of tropical diseases (TDs). The history of TDs dates back to ancient times, including the Roman and Egyptian empires. TDs are defined as diseases that are prevalent or indigenous to tropical and subtropical regions. Some of the most common TDs include malaria, cholera, yellow fever, dengue, Zika, etc. [1,2].

Mosquitoes are arthropods which are involved in the transmission of multiple pathogens, causing diseases that include dengue fever, chikungunya fever, malaria, filariasis, *Japanese encephalitis*, Zika, etc. TDs caused by mosquitoes have been associated with the mortality of humans every year. Therefore, mosquitoes are a major public health threat and thus can affect the economies of infected regions or countries. In the past few years, synthetic pesticides have been used to control mosquitoes. However, synthetic pesticides can cause contamination, kill many beneficial insects, and lead to the development of resistant-types after long-term use [3].

Advances in microscopy, molecular biology, biochemistry, diagnostic techniques, and treatment approaches in the 20th and 21st centuries have contributed to the understanding of the genetic contents of pathogens, biochemical reactions, and controlled such as susceptibility and resistance to drugs, vaccination of tropical diseases. The use of computer-aided techniques and other relevant technologies continue to aid experts in mapping regions of origins, predictions of spread, and creation of awareness using media outlets [2,4].

Early diagnosis of TDs is crucial for timely treatment, increasing patients’ survival rates, preventing further outbreaks, and minimizing the cost of diagnosis. Advances in science and technology continue to improve the accuracy, sensitivity, and specificity of diagnostic approaches. Currently, molecular testing and antibody-based approaches are regarded as the standard approaches for diagnosing TDs. Other techniques used by medical experts include imaging approaches (such as X-ray, CT scans, ultrasound), blood tests, microscopy, sputum tests, etc. These techniques have several limitations which include the need for sophisticated devices, the need for highly trained and skilled pathologists and medical laboratory technicians, the use of toxic chemical reagents, and a lack of POC diagnostics [4].

Genome editing technology is regarded as one solution that can be used to modify the genome of organisms and harness their mechanism as a form of biomimetic approach for accurate detection of diseases. The three most widely techniques employed for manipulating the genomes of different species, including mosquitoes, include Zinc-finger Nucleases (ZFNs) and Transcription Activator-like Effector Nucleases (TALENs). Advancement in molecular biology has led to the discovery of unique immune pathways utilized by bacteria to fight against viruses such as bacteriophages [5,6]. This immune response approach is termed CRISPR (Clustered Regularly Interspaced Short Palindromic Repeat). When viruses invade bacteria, they inject their nucleic acid (in the form of RNA or DNA) which hijack the bacterial DNA replication system, generating more viruses and subsequently destroying the bacterial cell. To prevent this type of invasion, bacteria utilize a three-step process that includes adaptation, expression (biogenesis or recognition), and interference to ensure immunity [7].

Since the first deployment of CRISPR/Cas9 for genome editing in 2012, many researchers have successfully applied this technique to accurately edit the genomes of a variety of organisms such as bacteria, yeast, plants, and animals including mosquitoes. CRISPR/Cas technology is cheaper, easy-to-use, and accurate compared to ZFNs and TALENs. Scientists biomimic this pathway by designing a synthetic RNA known as single guide RNA (SgRNA) which binds with the target (pathogen) nucleic acid (RNA in viruses and DNA in bacteria and parasites). The application of the CRISPR/Cas system has proven to be among the most, reliable, accurate, sensitive, specific, and fast methods for screening pathogens associated with TDs. CRISPR/Cas9 genome editing technology also enables the modification of the target genes of pests. This is especially useful in controlling vector-borne diseases caused by mosquitoes [3,6,8].

### Scope and Contribution

Throughout history, TDs have caused health issues around the world and contributed significantly to the mortality rate as well as the socioeconomic status of infected regions. Among numerous TDs, diseases associated with mosquitoes as carriers are the most common types of TDs with a high incidence and death toll. Despite measures taken by international organization such as the WHO, UNICEF, Red Cross, etc., to mitigate the array of infections cause by mosquitoes, progress is hindered by several factors such as resistance to insecticide, the drug resistance of parasites, climate change, urbanization, deforestation, lack of awareness, and lack of standard medical resources and social amenities in rural and underdeveloped regions. Early diagnosis is the first line of action in terms of the management of disease. In recent years, scientists have developed several laboratory assays for the rapid screening and detection of pathogens. Despite the progress achieved, current approaches are still hindered by factors such as low sensitivity, specificity, accuracy, false positive results, and misdiagnosis. The discovery of CRISPR/Cas systems in prokaryotes has open the window for scientists to repurpose or biomimic this approach in living cells. The CRISPR toolbox is a Pandora’s box that has several applications which include genetic modification (knock-in and knock-out of genes) and biosensing technology [6,8].

Thus, this review is focused on providing an extensive knowledge of TDs in terms of their pathology and epidemiology, with more focus on mosquito-causing diseases. Consequently, the review provides background knowledge on the mechanism of CRISPR/Cas systems in prokaryotes, classification of Cas systems, and the application of CRISPR as a gene editing tool. The application of CRISPR/Cas systems has been shown to aid in disease diagnosis and treatment and the generation of Genetically Modified Organisms (GMO) in both plants and animals. Therefore, this review discusses the use of this technology in biosensing and disease detection.

The remaining part of the review is as follows: Section 2 overviews the concepts of TDs and classification of TDs based on pathogens (which includes bacteria, viruses, and parasites). Section 3 presents the discovery of CRISPR/Cas systems in prokaryotes (based on adaptation, expression, and interference) and the classification of Cas systems. Section 4 overviews the concept of the CRISPR/Cas gene editing tool. Section 5 provides the up-to-date literature on the application of CRISPR/Cas-based biosensors for the detection of TDs. Section 6 presents the open research issue and provides conclusions.

## 2. Tropical Diseases (TDs)

The study and classification of TDs became a hot topic during the era of exploration and colonialism by the British, American, Portuguese, Spanish, French, etc., who came in contact with these type of diseases in tropical regions. The study, diagnosis, and treatment of these diseases led to the establishment of tropical medicine. Increased research in this field has led scientists to understand the mode of transmission, vectors, and symptoms of these diseases during the 19th century. Moreover, pathogens such as viruses, bacteria, fungi, and parasites that are associated with TDs were identified, as well as vectors such as lice, mosquitoes, fleas, etc., and other TDs associated with food and water contamination [2,4].

Many TDs spread as a result of interactions and complex cycles of transmission between human primates and animals such as invertebrates (e.g., flies, mosquitoes, snails) and vertebrates (e.g., livestock, dogs, cats, bats, snakes, etc.). The widespread and reemergence of TDs depends on several factors including an increase in population or population growth, global warming, exploration, migration, deforestation, meteorological events such as flooding, urbanization, etc. However, a change in environmental conditions such as extreme weather conditions and variations in rainfall, temperature, and humidity have influenced the widespreadness of TDs compared to other factors. Variations of rainfall and temperature have both been associated with influencing pathogen and vector replication and reproduction, as well as vector metabolism, host distribution, and the selection of habitats for breeding [9].

### 2.1. Transmission of Tropical Diseases

In both tropical and temperate climate regions, numerous viral and bacterial diseases are spread via several routes including transmission from one person to another through coughing, sneezing (airborne disease), or sexual contact (sexually transmitted diseases). Example of airborne diseases include tuberculosis, measles, and respiratory syncytial virus. TDs can be also be transmitted through drinking contaminated water and food sources (also known as waterborne and foodborne diseases, respectively). The mechanism of transmission of the majority of these diseases depends on an intermediate carrier also known as a vector. These organisms or carriers harbor these pathogens from an infected person or animal (zoonotic) and transfer it to others. Most often, these pathogens undergo mutation or developmental changes within the carriers which make them more virulent and difficult for the human immune system to fight [9,10].

### 2.2. Classification of TDs

There are several ways in which TDs can be classified. However, the most common classifications are based on the type of pathogen (such as viruses, bacteria, parasites, etc.), vectors, or carriers (such as ticks, mosquitoes, flies, etc.), which are also termed as arthropod-borne diseases and also based on concern (neglected and non-neglected TDs) [2,4]. When these diseases are transmitted by arthropods (such as flies, ticks, or mosquitoes), they are termed arboviruses or arthropod-borne viruses [2].

#### 2.2.1. Viruses

Virus-causing diseases are one of the most common and widely distributed pathogenic diseases in nature. Unlike bacteria that store their genetic content in the form of DNA, viruses store their genetic constituent in the form of RNA. They invade and hijack the host’s nucleic acid replication system which provides the necessary machinery to replicate new viral particles [11].

##### Dengue Virus (DENV)

Dengue fever is a disease caused by positive-stranded RNA containing the virus known as dengue virus, which is transmitted by a mosquito-borne flavivirus. *Aedes aegypti* mosquitoes are regarded as the main carriers of dengue virus and can transfer this virus during feeding on human primates (also known as Human-to-Mosquito Transmission). Moreover, medical experts also report the possibility of maternal transmission (from pregnant mothers to their babies). The most common acute symptoms of dengue fever include severe pain in the muscles, joints, ocular inflammation, headache, nausea, vomiting, rashes, swollen glands, etc. When this virus infects infants and children, it causes “dengue hemorrhagic fever” which leads to critical conditions such as shock (also known as “Dengue shock syndrome”) and circulatory system failure. Despite the prevalence of DENV around the world, there is no specific medication against the virus. However, doctors control the disease using medications to lower fever, relieve pain, prevent dehydration, manage bleeding, etc. [12].

In terms of epidemiology, DENV is found in many tropical and subtropical areas around the world and has been reported in more than 100 countries including Africa (Ivory Coast, Seychelles, Reunion Island, Cape Verde, etc.), Asia (Bangladesh, Afghanistan, China, Cambodia, Indonesia, India, Pakistan, Malaysia, etc.), America (Brazil, Peru, Ecuador, Nicaragua), and Australia. DENV has caused several endemics and epidemics around the world, with the incidence of the virus having recently increased due to human factors such as deforestation, massive urbanization, and global warming, which has expanded the regions inhabited by the Aedes mosquito vector. Approximately 400 million cases and more than 20 thousand deaths are reported almost every year, with more than 3 billion people at risk. The first outbreak of DENV dates back to 1779 in Indonesia and Egypt [13]. The disease was also recorded in North America in 1780 and it has reemerged over the years. In 2010, more than 1.5 million cases of DENV were reported in both South and North America. However, the largest number of infected cases was reported in 2016 in the United State of America (USA), with more than 2.38 million cases [14]. Several countries continue to report an increased number of cases daily, with Brazil having the highest number with more than 167 thousand as of March 2022.

##### Zika Virus (ZIKV)

ZIKV is another mosquito-borne disease that is predominant in several tropical and subtropical areas of West Africa, East Africa, South America, and Asia. The virus is a single-stranded positive-sense RNA virus that belongs to the “*Flaviviridae*” family. ZIKV shares numerous characteristics with other flaviviruses such as DENV, yellow fever virus, West Nile virus, and Japanese encephalitis. Aedes mosquitoes are regarded as the main carriers of ZIKV and can transfer this virus during feeding on human primates. During feeding, the virus is injected by mosquitoes which further replicates in dendric cells and is subsequently transported in the blood to other organs and tissues [15,16].

The virus can be acquired in the laboratory, through sexual intercourse or blood transfusions, or via the exchange of other bodily fluids such as breast milk, saliva, or the urine of an infected patient. The vector-borne transmission of the virus occurs in two cycles known as the sylvatic and urban cycles. The sylvatic cycle revolves around transmission of the virus by arboreal mosquitoes to non-human primates (NHPs), while the urban cycle revolves around transmission between human primates and urban mosquitoes. Scientists have also identified the virus antibodies in animal species such as goats, sheep, buffalo, lions, elephants, zebra, hippos, etc. The virus has been associated with Guillain-Barre syndrome in adults and microcephaly, arthrogryposis, ophthalmological defects, hearing defects, and cerebral malformations in children. The majority of infections caused by ZIKV are asymptomatic [16,17].

In terms of epidemiology, the virus was first isolated from the sentinel rhesus monkey in Uganda in 1947, while the first human isolation of the virus was reported in Nigeria in 1952. Since then, the disease has caused several epidemics and endemics around the world. Despite ZIKV having caused several health burdens, it was not until 2016 that the WHO declared it as a global health emergency due to the outbreak of the disease in South America. Just like many pathogenic tropical viruses, there is no specific antiviral drug or vaccine against ZIKV. Almost 100 thousand cases were reported in 2016, 609 in 2017, 1800 in 2018, and 15 cases in 2019 using EpiWATCH [18].

##### Yellow Fever Virus

Yellow fever virus is from the *Flaviviridae* family which causes yellow fever. It is related to *Japanese encephalitis*, St. Louis encephalitis, and West Nile virus. Yellow fever viruses are transmitted to people through a carrier known as Haemagogus or *Aedes mosquitoes*. These mosquitoes acquire the virus through feeding on infected animals or humans and transmit it to other primates. Thus, people infected by yellow fever virus through *Aedes mosquitoes* are referred to as being “viremic”. Yellow fever has three transmission cycles which include sylvatic (jungle), where the virus is transmitted by mosquitoes from NHPs, such as monkeys to humans visiting the jungle; savannah (intermediate), where the virus is transmitted from mosquitoes directly to humans (human to human); or from NHPs to humans. Urban transmission is initiated by viremic humans who have visited the savannah or jungle region and urban mosquitoes which feed on the infected person and transmit the disease to other humans [19,20].

Some of the mild symptoms associated with this disease include headache, fever, chills, back pains, weakness, fatigue, vomiting, and nausea. When left untreated, it can lead to critical or severe conditions such as liver, kidney, and heart failure or malfunctions, shock, jaundice (yellow skin), bleeding, etc. The mortality rate is high, as more than 50% of people infected with the virus die of the disease. There is no specific drug against yellow fever diseases; however, physicians prescribe medications that relieve pain, fever, and aches [21].

Unlike DENV that is prevalent in almost every continent, yellow fever is limited to Africa where it originated and has caused several epidemics in South Africa and other African countries such as the Democratic Republic of Congo (DRC) and Angola [20]. Several incidences of the disease have also been reported in Latin America, with more than 12 South American countries affected. Despite the fact that there are vaccines against the disease, the prevalence of the disease continues to spread, resulting in more than 70 thousand deaths per year. The increase incidence of the disease is associated with the widespread distribution of *Aedes mosquitoes* as a result of climate change [22]. Yellow fever has been recognized as a disease of significant public concern due to it pathology and high mortality rate in both human and NHPs. However, little is known about why the cases cease in some years and appear in other years, and what promotes the strong seasonal trends [21].

##### Rotavirus

Rotavirus is a pathogenic double-stranded RNA virus from the *Reoviridae* family. The name “Rota” is derived from the Latin word meaning “wheel”. Rotavirus is regarded as one of the most common pathogens that are detrimental in terms of pathology and is associated with causing watery diarrhea in children under 5 years of age. Other symptoms of the rotavirus-causing disease apart from severe dehydration include fever, nausea, vomiting, etc. Human primates are the reservoir of the disease which is found in the gastrointestinal tract and stool. The disease can be transmitted from person to person through fecal–oral routes (i.e., injecting infected food or water) and fomites (environmental surfaces contaminated by the stool of infected patients). The incidence of rotavirus is also reported in NHPs such as mammals, e.g., pigs [23,24].

The history of rotavirus dates back to the 1970s when several pediatricians and other medical experts embarked on studies to explore the causes of diarrhea in children as a result of striking mortality rates ranging from 3–12 million per year. Regions of high incidence include Bangladesh, Peru, and Guatemala which recorded more episodes of diarrhea cases. As a result of research using instruments such as electron microscopy and other biomedical instrumentation, scientists discovered several viral-causing diarrheas such as Norwalk agent and rotavirus (which appears to be a wheel-shaped virus), as well as other causing pathogens including different species of bacteria and parasites [23,24].

Globally, more than 2.7 million incidences are recorded and 600 thousand children die as a result of the virus annually, with the majority of deaths reported in India (i.e., with more than 100 thousand deaths yearly). Moreover, the majority of cases are reported in sub-Saharan Africa and South-East Asian countries. It is estimated that the disease leads to more than 500 thousand deaths in most of the underdeveloped countries. In the US, rotavirus is regarded as the most common cause of severe gastroenteritis in children [23,25].

Scientists over the years have developed vaccines against the virus, with the first developed in the USA in 1998 known as Roatshield, which was later withdrawn due to rare adverse effects. Currently, there are several vaccines used to treat rotavirus (such as Rotavac, Rotateq, Rotasil, Rotarix, etc.), in more than 100 countries around the world. The increase in vaccination has led to a decrease in incidence, hospitalization, and mortality among infants. Despite the massive level of vaccination, the disease continues to cause concern in many underdeveloped countries with low access to vaccines and quality medical care [24].

##### Human Immunodeficiency Virus

HIV/AIDS is one of the most common diseases that affects the immune system. It is caused by human immunodeficiency virus which belongs to the genus of viruses known as “*lentiviruses*”. In terms of its pathophysiology, HIV has been described to overcome or overpower the immune system’s T-cells known as CD4 helper cells, rendering the immune system susceptible to invasion from other pathogenic agents and cancers. As a result of the decline in response to foreign invaders by the immune system, HIV is accompanied by the term AIDS (Acquired Immune Deficiency Syndrome). The virus can be transmitted in several ways as a result of the exchange of bodily fluid such as blood, breast milk, vaginal secretions, and semen. The virus can also be transmitted from an infected pregnant mother to her baby. Some of the mild symptoms of the disease include fever, headache, sore throat, rashes, diarrhea, cough, swollen lymph glands, weight loss, etc. [26,27].

In terms of epidemiology, HIV is believed to originate from West Africa where it was transmitted to humans by a subspecies of chimpanzee. The disease is among the list of the most critical diseases that have emerged in the history of humanity. By 1996, the disease had already infected more than 13 million people within sub-Saharan Africa. The disease was declared epidemic in 1989 by the WHO as a result of an increase in the number of cases [28]. Despite major advances in diagnosis and treatment of HIV over the past two decades, it still remains a global concern. Currently, there is no specific drug against the virus. Even though there has been a decrease in the number of deaths cause by the virus, it is still prevalent in poor countries with substandard medical care systems. HIV has spread to almost every country and it was estimated that more than 37 million people were living with the virus in 2020 [29].

##### Ebola Virus

Ebola virus is one of the reemerging diseases causing severe health issues in African countries. It is formerly known as Ebola hemorrhagic fever. Symptoms of the disease cause by the virus include fever, hemorrhage, headache, and vomiting diarrhea. The mode of transmission of the disease is still unclear but medical experts believe the virus can be acquired as a result of direct contact with bodily fluid such as bloods and other secretions from infected patients as well as contact with surfaces contaminated with the virus. Scientists have categorized it as zoonosis and linked the disease with fruit bat and porcupines [30,31].

Ebola virus originated from two African countries, including the DRC and Congo, in 1976. The virus reemerged in West African countries, with early incidences in Liberia, Guinea, Sierra Leone, and Nigeria in 2014, and is regarded as the most serious health emergency crisis in the region. As of 2015, there had been more than 28 thousand reported cases and more than 11 thousand deaths. The average fatality rate of the disease is 50%; however, fatality rates in the past outbreak have varied between 25–90%. Even with advances in the diagnosis of the disease using advance technology, the outbreak of Ebola still remains intermittent and unpredictable. There have been more than 30 outbreaks of Ebola since 1976 [30]. The two recent outbreaks were reported in the DRC in 2018, with more than 3 thousand cases, and on 7 February 2021 [31,32].

#### 2.2.2. Bacteria

Bacteria are among the most abundant and ubiquitous microbes in nature. Bacteria can be classified as either pathogenic or non-pathogenic (e.g., microbiomes). They can also be classified as Gram positive or Gram negative. Some of the pathogenic bacteria that cause diseases include *Mycobacterium tuberculosis*, *Vibrio cholerae*, *Escherichia coli*, etc. [33].

##### Tuberculosis (TB)

TB is one of the most common bacterial diseases caused by bacteria know as *Mycobacterium tuberculosis*. It was discovered in 1882 and its mode of transmission was first reported in 1909. It is a slender, rod-shaped microbe with length ranging from 1–10 mm and strict aerobes (i.e., needing oxygen to survive). Tuberculosis is an airborne disease that is transmitted from an infected patient to others via sneezing, coughing, talking, etc. Depending on the environment, the bacterial particles can remain suspended in the air for hours and thus can be transmitted as a result of coming in contact with surfaces contaminated with the bacilli [34].

The pathogenesis of the bacteria occurs in the lung’s alveoli where it causes pulmonary tuberculosis. A few weeks after exposure, a granuloma is formed as a result of the immune system response against the bacilli. When the bacteria spread to other tissues in the body it is termed as “systemic miliary tuberculosis”. Treatment of tuberculosis depends on the severity of the disease. Pulmonary tuberculosis is mostly treated using antibiotics. However, *Mycobacterium tuberculosis* is becoming resistant to drugs and thus has increased virulence [35].

TB still remain a global health issue despite the use of antibacterial drugs against the bacteria. As of 2015, there are more than 10 million people suffering from the disease, with 10% mainly children and 12% people suffering with HIV/AIDS. As of 2015, the number of deaths associated with TB was estimated to be around 2 million. In the last few years, cases of TB have declined marginally. Even though the disease has been controlled in many countries, it is still a health issue in many underdeveloped countries and continues to threaten to become an increasing burden due to both extensive drug resistance and multi-drug resistance [35].

##### Cholera

Cholera is another bacterial disease that is associated with diarrhea. It is caused by a Gram-negative bacterium with a coma-like shape known as *Vibrio cholerae*. Cholera can be transmitted through the fecal–oral route as a result of eating food or drinking water contaminated with the bacteria. When the bacterium enters a host cell, it secretes toxins which leads to symptoms such as diarrhea, vomiting, abdominal pain, and hypovolemic shock. Factors that increase the risk of the disease include lack of access to clean and sanitized water, people with O blood group, living in overcrowded societies, use of antihistamine and proton pump inhibitors, etc. [36,37].

Despite progress in research regarding the diagnosis and treatments of diseases, cholera continues to be a burden in many countries. In terms of outbreaks, scientists have identified two serotypes known as O1 and O139 which causes disease, while more than 190 serotypes are non-pathogenic. O1 has been associated with the most recent outbreaks in Bangladesh and Kerala, India. O139, on the other hand, has caused sporadic outbreaks in some regions within Asia [37,38]. Despite the fact that the majority of positive cases and deaths toll are underreported, it was estimated that there are more than 4 million cases of cholera yearly and more than 140 thousand deaths globally. The disease is found to be endemic in many countries within Asia and Africa, while cases have been reported in countries within the Caribbean, Middle East, and South and North America [38,39].

##### *Escherichia* *coli*

*E. coli* is another bacterium that causes bacteremia in some developed countries. The bacteria are found in the lower intestine of blooded animals. There have been several strains of *E. coli* identified and the majority do not cause infection, while a few are pathogenic and have been found to cause food poisoning and diarrhea. An example of this bacteria is the Shiga toxin-producing *E. Coli* O157:H7. This strain is an enterohemorrhagic type that causes diarrhea hemolytic-uremic syndrome and hemorrhagic colitis in humans. It is classified as both a food and water-borne disease that is transmitted via the fecal–oral route as a result of consuming uncooked meat and contaminated liquid, including raw meat and vegetables [40]. Pathogenic *E. coli* are known to cause travelers’ diarrhea and kidney problems. Common symptoms include abdominal cramp, vomiting, fever, and diarrhea. Outbreaks of this pathogenic bacteria have been reported in Japan, the USA, and Scotland [41].

#### 2.2.3. Parasites

Parasites are group of organisms that live in or on another organism known as the host, at whose expense they obtain their nourishment while simultaneously infecting the host. Parasites can be classified as single-cell organisms (e.g., protists) and multi-cell organisms (e.g., helminths or worms). Parasites ranges from micro-size to macro-size organisms. Even though some parasites can be found intracellular, the majority live extracellular inside the host and are mostly found in the gut, blood, lymphatics, etc. Unlike bacteria and viruses that replicate inside host primates, parasites undergo complex developmental transformation within the host and can reproduce sexually and asexually. Examples of parasitic diseases and pathogens include *Plasmodium* (malaria), *Trypanosoma* (African sleeping sickness, Chagas disease), hookworm (ancylostomiasis), roundworms (Ascaris), tapeworm (Dipylidium caninum disease), *Leishmania* (Leishmaniasis or kala-azar), etc. [42].

##### Malaria

Malaria is the most common parasitic disease globally. It is cause by protists known as *Plasmodium*. There are different species of *Plasmodium*; however, *Plasmodium falciparum* is identified as the most virulent and the leading cause of death among *Plasmodium* species. *Plasmodium* is transmitted to human primates by anopheline mosquitoes during feeding. After injection of the parasites by the carrier, the infection cycle begins in the liver cells followed by the red blood cells where the parasites consume hemoglobin. The completion of the cycle ends in the erythrocytes, where the parasites divide and infect more red blood cells. The general symptoms of malaria include fever, weakness and fatigue, sweating, nausea, vomiting, diarrhea, headache, abdominal pain, etc. [43,44].

Malaria is more prevalent in sub-Saharan Africa countries, where there are widespread untreated water bodies. Malaria cases are estimated to be around 300 million with more than 1 million deaths every year. Malaria has also reemerged in countries that had been declared malaria-free. Outbreaks of malaria continue to be an issue in Africa, the Amazon region, and Asia. The WHO and other international organizations have launched several campaigns and projects to eradicate malaria globally but it still remains elusive. Factors associated with persistent malaria include resistance to insecticide and drugs, lack of adequate healthcare facilities and sanitation programs in underdeveloped countries, and lack of priority concern from international bodies [45,46].

##### Trypanosomiasis

Trypanosomiasis is another disease that is cause by species of the genus Trypanosoma. An example of trypanosomiasis is Chagas disease, also known as American trypanosomiasis that is cause by *Trypanosoma cruzi*. Unlike malaria, trypanosomiasis is transmitted by bugs which feed on infected feces and enter into human primates through the mouth, nose, skin, eyes, etc. Symptoms of this disease include inflammation or swelling of the lymph nodes and fever, while in critical conditions it can lead to cardiac malfunction, digestive disorders, and death [47].

Another common disease-causing trypanosomiasis is known as sleeping sickness or African trypanosomiasis, which is transmitted by tsetse flies (Glossina species). Species associated with this disease include *Trypanosoma brucei rhodesiense*, which is found in South and Eastern Africa, and *Trypanosoma brucei gambiense,* which is most common in Central Africa and West Africa. The common acute symptoms of African trypanosomiasis include weakness, dizziness, fever, and headache. In a critical condition, the disease can cause neurological disorders with symptoms such as delusions, hallucination, seizures, etc. [48,49]. Epidemics of sleeping sickness have caused concern in the past. However, as a result of intervention by both national and international organizations, the disease is well-controlled with cases of less than 600 reported in the DRC in 2020 [50].

##### Leishmaniasis

Leishmaniasis is a parasitic disease that is cause by protists known as the Leishmania genus. Scientists have identified about 20 species that causes disease to human primates and NHPs (i.e., mammals). An example of Leishmaniasis is Cutaneous Leishmaniasis, which is locally known as oriental sore, Delhi boil, or Baghdad ulcer. Another example is Visceral Leishmaniasis, which is locally known as kala-azar in India and refers to black sickness. In recent years, the number of cases has surged from less than a million to 1.2 million. Visceral Leishmaniasis is regarded as the most dangerous form of Leishmaniasis. Leishmania can be transmitted as a result of a bite from infected phlebotomine sandflies. This species is found within the macrophages and plays a crucial role in fighting against invading microorganisms in the host’s body [51]. The symptoms of Leishmaniasis include weight loss, increase pigmentation, fever, and swelling of the liver and spleen. Apart from humans, other reservoir hosts identified include dogs and rodents [51,52].

In terms of epidemiology, the disease is prevalent in both tropical and subtropical regions, with recent cases in Sudan and India. Few cases have been reported in the US and Southern Europe, while Australia and Antarctica are the only continents with no reported cases. According to the WHO, there are more than 10 million recorded cases with 300 million people at risk in more than 90 countries. The disease is predominant in rural settlements but can also be found in the outskirts of cities [53].

### 2.3. Neglected Tropical Disease (NTDs)

The last century has witnessed a decline in the incidence of and elimination of numerous TDs in the majority of developed countries. However, millions of people are still affected by these types of diseases, especially in underdeveloped countries which contribute to high mortality rates. These types of diseases are termed as NTDs. Example of NTDs include leprosy, Guinea worm disease, African sleeping sickness, rabies, Leishmaniasis, Schistosomiasis, Fascioliasis, dengue, Dracunculiasis, Onchocerciasis, Chagas disease, yaws, hookworm, trachoma, etc. [54].

The prevention and control of NTDs in underdeveloped countries are highly challenged by several factors such as the socioeconomic status of the regions, a lack of medical equipment, a lack of adequate response and concern from international organizations, a lack of awareness, etc. NTDs can be found in some regions located within Africa, Asia, and Latin America. The majority of NTDs are associated with rural areas and regions which lack access to hygienic food, clean water, and safe ways of waste disposal [32].

The primary ways of controlling or preventing NTDs include controlling the vectors or via massive drug administration. As carriers of pathogens, vectors play crucial role in disease pathways. Thus, controlling vectors such as black flies and mosquitoes that transmit disease as well as improving environmental hygiene and water sanitation are highly crucial for controlling and preventing NTDs. Consequently, massive drug administration is another effective way or intervention in eliminating NTDs. Diseases that can be eliminated using this intervention approach include trachoma, Onchocerciasis, Dracunculiasis, Schistosomiasis, lymphatic filariasis, and soil-transmitted helminths (hookworm or Ascaris) [55].

## 3. CRISPR in Prokaryotes

The CRISPR systems along with Cas proteins are highly diverse adaptive immune mechanisms used by many bacteria and archaea to protect themselves from attacks by viruses, plasmids, and other foreign nucleic acids. CRISPR consists of short, highly conserved repetitive sequences (23–44 bp long) separated by spacers. These spacers are unique sequences and are usually derived from phages or the plasmid’s DNA. This adaptive system can learn to recognize and cut specific NA regions of invading pathogens and store them [56].

CRISPR evolved as an immune response or mechanism against phages. The basic mechanism of the CRISPR/Cas system arises from the need to obtain viral DNA or RNA, with most archaea (~87%) and bacteria (~47%) being clustered in normal short intervals. Intact CRISPR loci within the genome include a series of CRISPR arrays, CRISPR-related protein (Cas) genes, and direct repeats separated by multiple spacers. With the onset of the virus, the CRISPR/Cas locus triggers a three-step immune response, “adaptation–expression–interference”, which destroys phages that invade the host cell [6].

**Figure 1:**
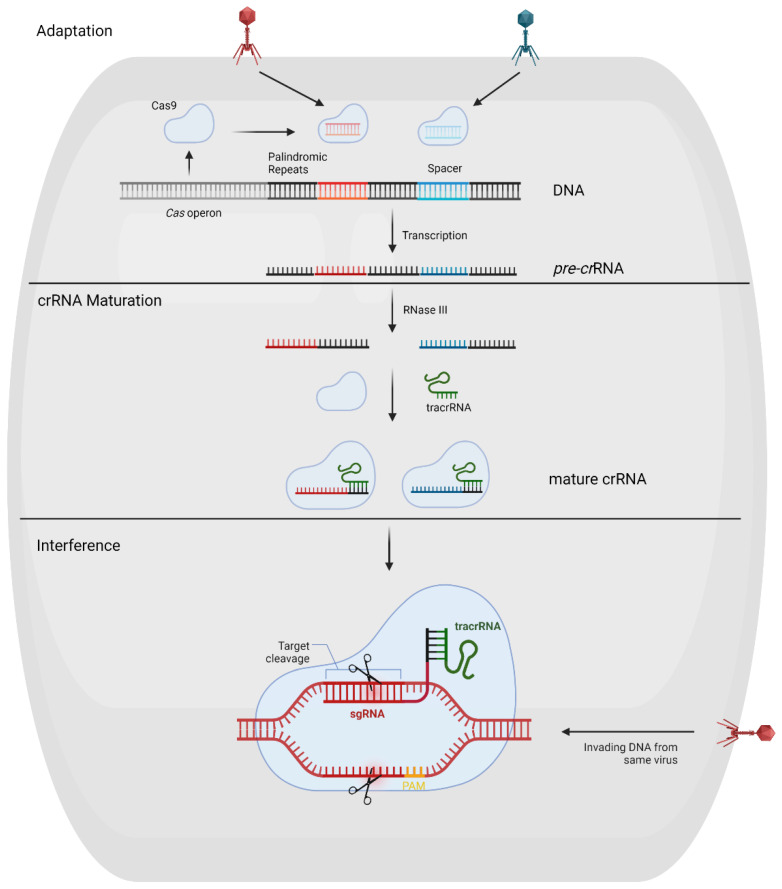
Schematic diagram of the CRISPR adaptive immune system against bacteriophage. (Adapted from Ref. [61] with free permission. Created with BioRender.com accessed on 20 September 2022).

Technically, the CRISPR/Cas system has only two components: (I) Cas protein, DNA, or RNA cleavage protein that promotes adaptive immunity in the process of adaptation, expression, and interference in prokaryotic cells, and (II) for cleavage of a target nucleic acid (DNA or RNA) as a known RNA molecular guide RNA which is programmed to navigate the system to recognize, bind, and cleave target NA [57]. The adaptation stage occurs when bacterial or archaeal cells first come in contact with viral DNA. The CRISPR loci translate Cas genes into Cas proteins (Cas9, Cas2, and Cas1). These Cas proteins survey for the viral DNA, cut part of it (known as spacer), and store it in the CRISPR array’s leader strand. The second stage, known as the expression stage, is only initiated when the viral DNA attack again. The bacterial or archaeal cell’s CRISPR array transcribes its stored spacers into small non-coding RNA, known as pre-CRISPR RNA, which link with TracrRNA through base pairing and form hybrid RNA or matured CRISPR RNA. The CRISPR RNA is employed in the third stage, known as the interference stage, where it forms a complex with an effector Cas enzyme (such as Cas9 which is translated from the Cas genes adjacent in the CRISPR loci). This complex locates the viral DNA as a result of the unique Protospacer Adjacent Motif (PAM) sequence and destroys the viral DNA, leading to complete immunity, as shown in Figure 1 [58,59,60].

### 3.1. Classification of Cas Systems

The CRISPR-Cas system can be divided into two classes depending on the number of effector Cas used in the interference stage. Class I contains multiple Cas effector complexes. Class II requires only one Cas protein. Based on their properties, the CRISPR/Cas system class can be divided into several different types, which are further subdivided into subtypes corresponding to specific Cas proteins. Recent studies have shown that types I, III, and IV belong to class I, and types II, V, and VI belong to class II. Today, the CRISPR-Cas system offers new methods of biosensing with its ability to identify single-base mismatches in target nucleic acids. Many Cas effectors possess specific (cis-cleavage) and non-specific (trans-cleavage) nucleolytic activities. Type II Cas9, Type V Cas12a, Type VI Cas13a, and Type V Cas14 are widely used along with guide RNA (gRNA) complexes to target-specific DNA/RNA [62]. The classification of CRISPR/Cas systems is presented in Table 1.

#### 3.1.1. Cas9

Cas9 is a double-spin RNA-driven type II DNA cleavage protein. Only double-stranded DNA (dsDNA) is required as the catalytic substrate. An adjacent protospacer motif (PAM) is required for the target DNA. Unlike other Class II effector types, Cas9 uses Rnase III to process the transactivation precursor RNA (tracrRNA) and CRISPR RNA (crRNA) complex before binding to dsDNA. Mature gRNA begins with a nucleotide spacer (nt) 20–24, followed by a tracrRNA: crRNA double chain. gRNA is also chimeric and can form single-stranded guide RNA (sgRNA). Cas9, which has multiple domains, interacts with mature crRNA to stabilize the crRNA and change its conformation, facilitating the binding of the next target. In the presence of dsDNA, Cas9 first looks for the PAM sequence, then recognizes the seed region and forms Watson Crick base pairing between the target dsDNA and the spacer. The RuvC and HNH domains cleave target strands (TS) and non-target strands (NTS) (3 nt upstream of PAM) to introduce blunt-ended double-strand breaks (DSBs). As mentioned earlier, the cleaved product still binds to the Cas protein and is released very slowly. In addition, Cas9 catalytic deficiency (dCas9) is acquired by mutations in the nuclease domain, which retains only its DNA-binding ability [63].

#### 3.1.2. Cas12a/Cas12b

Cas12a/Cas12b produces mature crRNA and directs the Cas protein to bind to the target DNA. The length of the mature crRNA was 42 × 10^44^ nt. It begins with a 19 NT direct iteration sequence in which the 19th U base is strictly retained, followed by a 23–25 nt spacer. The interaction of Cas12 crRNA triggers the conformation switch of Cas12a, exposing the active site of RuvC. When dsDNA containing the PAM-rich T sequence at the 30-end is perfectly fitted to the crRNA spacer, it forms an R-loop with crRNA. NTS is placed in the active site of RuvC for the next cleavage. After cleavage, Cas12a allows the release of the truncated product, revealing the active site of RuvC. This produces a staggered DSB with a 5 nt overhang at the 50th edge. Interestingly, Cas12a’s transactivity allows it to cleave adjacent ssDNA without the need for a specific sequence. In contrast to Cas12b, dsDNA-triggered Cas12a has higher trans-cleaving efficiencies than ssDNA-triggered ones [61,62].

#### 3.1.3. Cas13a/Cas13b

Compared to other Class II Cas proteins, Cas13 showed cis and trans-cleaving activity against single-strand RNAs (ssRNAs). Cas13 interacts with (pre) crRNA to cause conformational change when it recognizes RNA with a 3′-protospacer flanking sequence (PFS, A/U/C). When the seed region closely matches the target from the center of the spacer, it then extends throughout the spacer to form a double chain. In addition, the HEPN domain approaches the construction of complex active sites on the outer surface of Cas13, causing Cas13 to function as a non-specific Rnase. Studies have shown that a 20-base pair (bp) guide target double-stranded RNA is essential for activation of the catalytic site of the HEPN domain. Furthermore, Cas13 lacks a specific cleavage site but shows a cleavage preference for U [62,64].

#### 3.1.4. Cas14

Cas14 is one of the most recent characterized Cas systems which shares similar traits with CRISPR/Cas type V. As a new member of the CRISPR effector, Cas14 is much smaller in size (40–70 kDa) compared to other Cas proteins in the Class II system and typically has a molecular size of 100–200 kDa. Unlike Cas9, Cas14 is regulated by tracrRNA (crRNA double chain or sgRNA). Unlike Cas13 that cleaves RNA, Cas14 cleaves single-stranded DNA targets similar to Cas12. Cas14 can recognize foreign DNA without the need for PAM sequences. In addition, Cas14 cleaves the target ssDNA beyond the spacer protospacer double chain region, and its collateral cleavage efficiency increases with ssDNA elongation. Cas14 also exhibits collateral cleavage activity against DNA, which makes it vital for the direct detection of pathogenic bacteria as well as RNA after reverse transcription. Despite the fact that Cas14 shares a lot of similarities with Cas12, Cas14 has a lower on-target as a result of sensitivity of the internal-seed sequence to nucleotide mismatch, as well as a lower tolerance to nucleotide mismatch that lies between the target template and sgRNA [65]. The differences between Cas effectors are shown in Table 2.

## 4. CRISPR/Cas as Gene Editing Tool

CRISPR/Cas9 has been shown to function as an adaptive immune system against viruses and phage through DNA binding by CRISPR RNA (crRNA) and DNA damage by Cas9 nuclease in bacteria. In genome editing, CRISPR/Cas9 functions with the help of a single guide RNA (sgRNA) that recognizes a target sequence (protospacer) in the genome of the host organism via complementary base pairs. The Cas9 nuclease then specifically creates a double-strand break (DSB) in the region close to the PAM sequence (Protospacer Adjacent Motif). A major advance in this area is the discovery of sgRNA. It was originally used in combination with Cas9 and made in vitro cuts at various DNA sites [3].

Unlike in prokaryotic cells, the CRISPR/Cas complex acts as an antiviral system to identify the genetic information of alien species (DNA or RNA fragments injected into the cell) and stores and shares it highly selectively and specifically. However, in the case of biomimicking the prokaryotic CRISPR/Cas mechanism in living cells, the guide RNA strand is synthetically designed to bind consistently with the DNA or RNA sequence of an exotic species. After finding the correct sequence, the CRISPR/Cas complex cleaves DNA or RNA into one or two nuclease domains (depending on the type of Cas protein) and creates nicks to make them single- or double-stranded [60].

**Figure 2:**
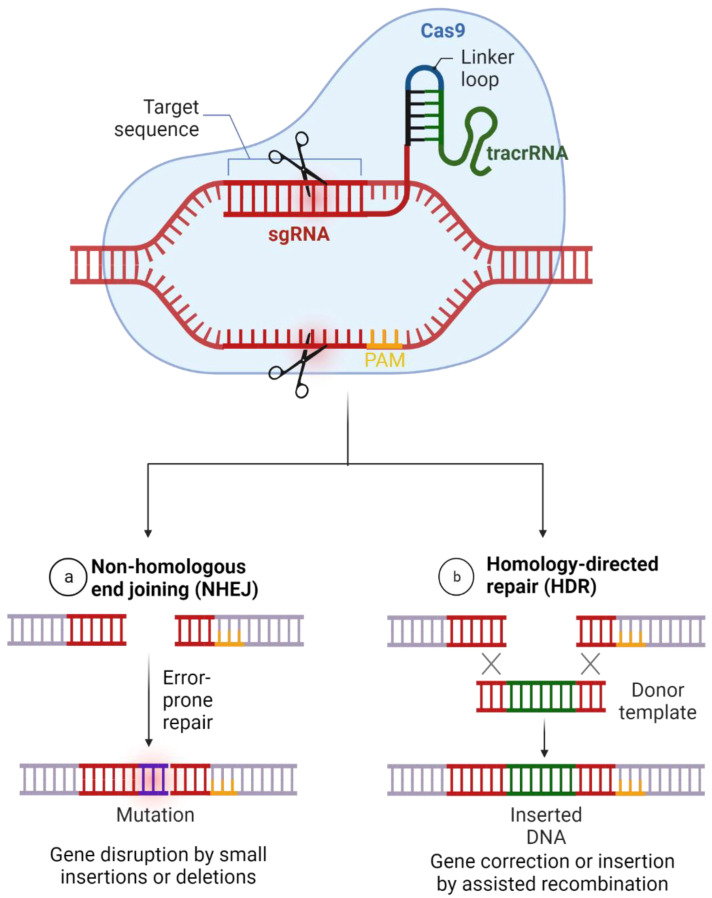
Gene Editing Using CRISPR/Cas system. (Adapted from Ref. [61] with free permission. Created with BioRender.com accessed on 20 September 2022).

Once the DNA/RNA is cleaved, the cells initiate a repairing mechanism known as Non-homologous End-joining (NHEJ), which is a natural way that cells stick together through insertions or deletions of nucleotides (known as indels). However, this method is prone to mutation and can lead to gene dysfunction or deactivation. Scientists can use this window to introduce a desired homologous DNA template through a process known as Homologous Repair (HR) or Homologous Directed Repair (HDR), as shown in Figure 2 [66].

### CRISPR/Cas9 in Parasites

The discovery of CRISPR/Cas systems has opened the gateway to several applications related to gene editing of parasites. Applications include the use of CRISPR/Cas9-mediated gene drive to interfere with vector transmission of parasitic diseases, the choice of selectable markers, novel delivery and treatment approaches, understanding of the pathogenesis of parasitic organisms through gene manipulation, etc. Despite the prospects of CRISPR/Cas9 gene editing technology, it is hindered by several challenges which include off-targets, gene mutations, and complex morphology and the life cycle of these parasites. Thus, there is a need to develop novel approaches that will increase the efficiency of CRISPR/Cas9 gene editing technology and improve on-targets, enhancing gene mutation efficiency and overcoming issues involved in the host passage [67,68].

Since the discovery of CRISPR/Cas9, the system has been used in a wide variety of bioscience and biomedical studies to edit genomes of a wide range of model organisms (which include *Caenorhabditis elegans*, *Saccharomyces cerevisiae*, *Drosophila melanogaster*, etc.), generation of animal models, cancer treatment, stem cell research, somatic genome editing, correcting genetic diseases, neurobiology, and the treatment of infectious diseases [68].

There are a handful of studies that attempted to knock-in or knock-out genes from parasitic organisms. Among these studies is the one provided by Dong et al. (2018) [69]. The study, as part of an approach to control mosquitoes, targeted the agonist’s journey of *Plasmodium* based on transmission-blocking using CRISPR/Cas9. The study proposed a CRISPR/Cas9 gene editing procedure which targeted a malaria vector known as *Anopheles gambiae* by inactivating fibrogen related protein 1 (FREP 1). The result of the study has shown profound suppression of malaria infection in adult mosquitoes (FREP1 knockout mutants). Another study that focused on controlling mosquitoes was provided by Macias et al. (2020) [70]. The study proposed an embryo injection method based on Receptor-mediated Ovary Transduction of Cargo (ReMOTE), which is used to transport Cas9 ribonucleoprotein complex to the ovaries of an adult *Anopheles stephensi*. The outcome of the study demonstrated the efficiency of ReMOTE in delivering Cas9 and the subsequent development of heritable mutations in adult mosquitoes.

Unlike the study conducted by [69] which focused on mosquitoes, the study conducted by Zhang et al. (2017) [71] revolved around *Plasmodium*. The study targeted *Plasmodium yoelii* ApiAP2 genes which have been shown to play a significant role in parasite development. The study identified 24 genes and 12 were successfully knocked out using CRISPR/Cas9. However, evaluation of the gene knockout in the development of *Plasmodium* in both mice and humans have shown that some of the genes are critical for the development of *Plasmodium yoelii*.

Gene drive technology is gaining attention due to it scalable impacts on controlling infectious diseases. The use of CRISPR/Cas9 is becoming the most important machinery for the genetic manipulation of parasites and vectors [72,73]. An example of gene drive technology for disease control was proposed by Hammond et al. (2016) [74]. The study identified three genes which contribute to recessive female sterility in mosquitoes. The genes are introduced into each locus using CRISPR/Cas9. The evaluation of the impacts of the genes in controlling the population of mosquitoes using population modeling and cage experiments revealed that one of the genes met the minimum requirements for gene drive.

The study conducted by Burle-Caldes et al. (2018) [75] applied the CRISPR/Cas9 gene editing approach for rapid generation of *Trypanosoma cruzi* gene knockout mutants. The study focused on the disruption of the GP72 gene, which is achieved either through transfecting wild type *T. cruzi* with recombinant *Staphylococcus aureus* Cas9 bind with guide RNA or through transfecting *T. cruzi* stably expressing *Staphylococcus pyogenes* Cas9 along with SgRNA. Due to the absence of NHEJ repair in the parasites, the study showed that gene knockout in *T. cruzi* occurs through HDR instead of microhomology-mediated end joining (MMEJ). Moreover, disruption of these genes has resulted in abnormal morphology and few parasites had their flagellum detached from their body.

The first reported editing of the CRISPR/Cas9 gene in kinetoplastids, for *Leishmania donovani* [76] and Leishmania major [77], used a method of expressing Cas9 from an episomal plasmid. Target-specific SgRNA targets are in vitro transfected sgRNA transfection or plasmid transfection for in vivo transcription of sgRNA from RNA Pol I [76] or RNA Pol III [77] promoters. Donor DNA for directed repair results in precise modification [76]. Cas9-induced double-strand cleavage was repaired by a microhomology-mediated end joining (MMEJ) mechanism without adding donor DNA, resulting in a small deletion at the target site [76]. The use of CRISPR/Cas9 for the knockout of the lipophosphoglycan (LPG) gene in *Leishmania* spp. was proposed by Beneke et al. [78] with a CRISPR/Cas9 toolkit for rapid and precise gene modification by integration of donor DNA, using engineered cell lines and drug selection of mutants. All required sgRNA and donor DNA cassettes are generated by PCR in just a few hours without time-consuming DNA cloning.

## 5. CRISPR-Based Biosensor

The NA detection technique is one of the molecular diagnostic approaches that has been trending over the past few years. Apart from PCR and RT-PCR that are established as good standard approaches for the detection of viruses and other pathogens, other approaches such as NA hybridization and isothermal application techniques have been developed for clinical diagnostics. Despite the viability of these approaches, they are hindered by several challenges such as low specificity, low sensitivity, and the need for laborious procedure, chemical reagents, and well-trained medical lab technologists [7,66].

CRISPR-based biosensors are gaining interest from scientists around the world due to their sensitivity toward target NA. The need to develop simple, robust, sensitive, accurate, cheap, and POC diagnosis for clinical applications is growing every year. CRISPR toolbox is a Pandora’s box with several Cas systems that allow scientists to delete and insert gene of interests. This remarkable feature allows scientists to develop CRISPR-based biosensors as a subsidiary of a NA-based biosensor that can either bind or cleave to target nucleic NA. Inside this Pandora’s box are CRISPR/Cas9, CRISPR/Cas12, and CRISPR/Cas13 [79,80]. Recent studies have shown that Cas14 can be used as a promising tool for diagnosis and biosensing [62].

### 5.1. CRISPR/Cas9 or dCas9-Based Biosensors

**Figure 3:**
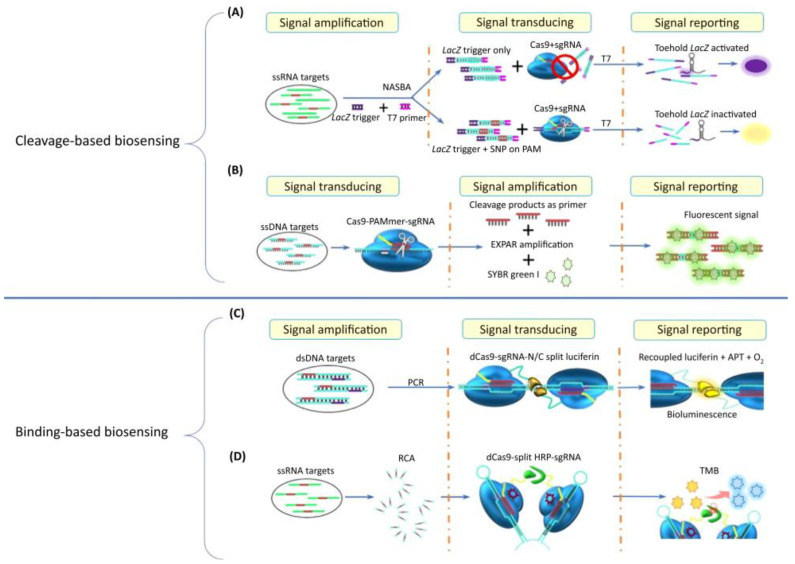
The use of CRISPR/Cas9 and dCas9 for the detection of diseases. (Reprinted with permission from Elsevier [79]. Copyright and Licensing.) The use of CRISPR/Cas9 for the detection of diseases can be classified into two approaches: (**A**,**B**): Cleavage-based biosensing. (**C**,**D**): Binding-based biosensing. (**A**). Nucleic Acid Sequence-based Amplification (NASBA)-CRISPR Cleavage (NASBACC). (**B**). CRISPR/Cas9 Triggered Isothermal Exponential Amplification (CAS-EXARP). (**C**). dCas9 conjugated to the split halves of luciferase, termed as paired dCas9 (PC) reporter. (**D**). Rolling Circle Amplification (RCA) for isothermal amplification of the target (microRNA), with dCas9 effectors fused together with split Horseradish Peroxidase (HRP).

The CRISPR/Cas9 is one of the most widely used Cas systems for gene editing. The process revolves around the use of a Cas effector along with single guide RNA which navigates through a matching target and cleaves it by inducing a double-strand break, as shown in Figure 3. The use of CRISPR/Cas9 for the detection of diseases can be classified into two approaches: (1) Cleavage-based biosensing (Figure 3A,B) and (2) Binding-based biosensing (Figure 3C,D). CRISPR/Cas9 has been harnessed along with other amplification techniques such as Nucleic Acid Sequence-based Amplification (NASBA)-CRISPR Cleavage (NASBACC) (as shown in Figure 3A) and CRISPR/Cas9 Triggered Isothermal Exponential Amplification (CAS-EXARP) (as shown in Figure 3B) reaction to form biosensing platforms for the detection of pathogens and discrimination between different strains. dCas9 is an inactive form of Cas9 which instead of cleaving the target, only binds to it. Scientists harnessed this feature to couple various modules such as split enzyme or fluorescent to develop a bioaffinity CRISPR-based biosensing platform [7].

Zhang et al. (2022) [81] developed a biosensing platform that use two pairs of dCas9 for the detection of *Mycobacterium tuberculosis*. The system was designed using pairs of dCas9 conjugated to the split halves of luciferase, termed as paired dCas9 (PC) reporter, as shown in Figure 3C. The Cas system was guided by two single guide RNA which activate luciferase. The binding of the complex generated highly intensified luminescent signals. Evaluation of the sensitivity of the biosensing system resulted in a sensitivity of 0.1 nM without DNA amplification and 35 aM with amplification using PCR (35 cycles).

The use of CRISPR-based optical Geno-biosensor for the detection of the Zika virus was developed by Pardee et al. (2016) [82]. The study employed CRISPR/Cas9 and isothermal RNA amplification which is able to discriminate between Zika genotypes with single-base resolution. The study also used DENV as a negative control. Evaluation of the optical Geno-biosensor has shown high specificity in discriminating between ZIKV and DENV. Moreover, the study conducted by Qui et al. (2018) [83] demonstrated the use of Rolling Circle Amplification (RCA) for isothermal amplification of the target (microRNA), with dCas9 effectors fused together with split Horseradish Peroxidase (HRP) protein to recognize and bind to the target in order to activate the colorimetric change of Tetramethylbenzidine (TMB), as shown in Figure 3D.

### 5.2. CRISPR/Cas12-Based Biosensors

Unlike Cas9, which possesses both RuvC and HNH domain which induce double-strand break, Cas12 only possesses RuvC domain which contributes to its single-strand break on target NA. Another difference between Cas12 and Cas9 is that Cas12 does not require Transactivating RNA and recognizes the target based on T-rich PAM sequence. An example of a biosensing platform that uses Cas12 is one-HOur Low-cost Multipurpose highly Efficient System (HOLMES). This system harnesses the cleavage activity of Cas12 with a quenched fluorescent to detect target DNA [84].

Integration of nanotechnology in biosensing technologies has been shown to improve sensitivity. The study conducted by Lee et al. (2021) developed a nanobiosensor for the detection of DENV. The system is designed based on Cas12 and methylene blue (MB) conjugated gold nanoparticles (MB-AuNPs) which increases the electrochemical factor. The performance evaluation of the electrochemical-based CRISPR biosensor exhibited 100 fM ultra-sensitive detection of DENV. One advantage of this system over several existing CRISPR-based biosensors is that it does not need the amplification step.

Wang et al. (2021) [85] developed a CRISPR-based nucleic acid detection platform known as Loop-mediated Isothermal Amplification coupled with CRISPR/Cas12a-mediated diagnosis (LACD) for the detection of *Mycobacterium tuberculosis*. The LACD assay comprised a LAMP amplification of the target DNA. The platform harnessed the trans-cleavage activity of Cas12a which cleaved target DNA. The degraded target DNA can be measured using a real-time fluorescence device or it can be visualized using a lateral flow biosensor. Evaluation of the sensitivity of the platform has shown that it can detect templates down to 50 fg of *Mycobacterium tuberculosis* Complex (MTC) genomic template per test.

Zhao et al. (2018) [86] developed an on-site biosensing technique for the detection of HIV. The sensor was designed based on hybridization between guide RNA coupled with Cas12a and target RNA which is amplified using Real-time Isothermal Reverse-transcription Recombinase-aided Amplification (rRT-RAA). The resulting cleavage of the target can be observed with the naked eye by using a blue light imager. Testing of the developed biosensing assay using clinical assay has shown that the system is capable of detecting 20 copies of purified HIV-1 RNA or DNA per reaction as low as 123 copies/mL of HIV-1 viral load.

The study conducted by Li et al., 2019 [87] developed Cas12b-based biosensor known as HOLMESV2. The biosensing platform harnessed the trans-cleavage collateral activity of the Cas system against target NA. The platform has shown excellent results in terms of discriminating single nucleotide polymorphism, detection of viral NA, human mRNA, and circular RNA. In order to avoid cross contamination and to amplify the target, the platform is designed along LAMP assay under constant temperature.

### 5.3. CRISPR/Cas13-Based Biosensors

Another remarkable discovery occurred in 2016 when Cas13 was discovered as a result of comprehensive research on the type VI CRISPR/Cas system. As discussed earlier, Cas9 possesses both RuvC and HNH domains and Cas12 possesses only RuvC domain. However, unlike both Cas12 and Cas9, Cas13 possesses special domains known as 2 Higher Eukaryotic and Prokaryotic Nucleotide (HEPN)-binding domains. Unlike Cas9, Cas13 possesses a special feature known as “collateral cleavage”. This special feature of Cas13 can be harnessed to cut label RNA reporters for the detection of nucleic acid from a different target which includes viruses, bacteria, and eukaryotic cells [88].

**Figure 4:**
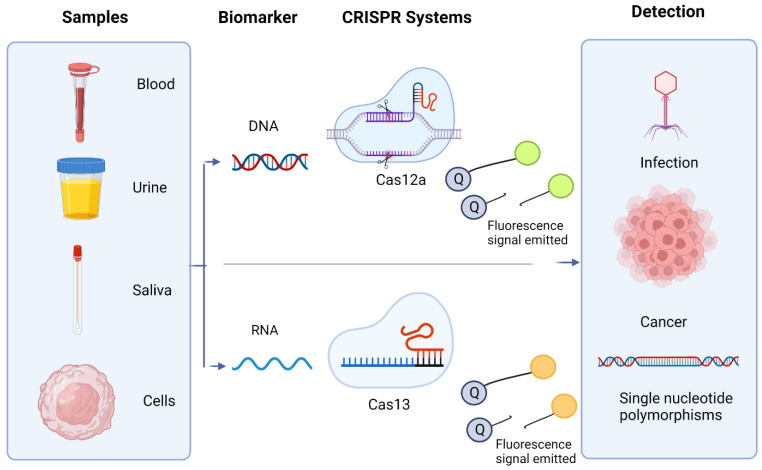
The use of CRISPR/Cas12 and Cas13 for detection of tropical diseases. (Adapted from Ref. [92] with free permission. Created with BioRender.com accessed on 21 September 2022).

**Figure 5:**
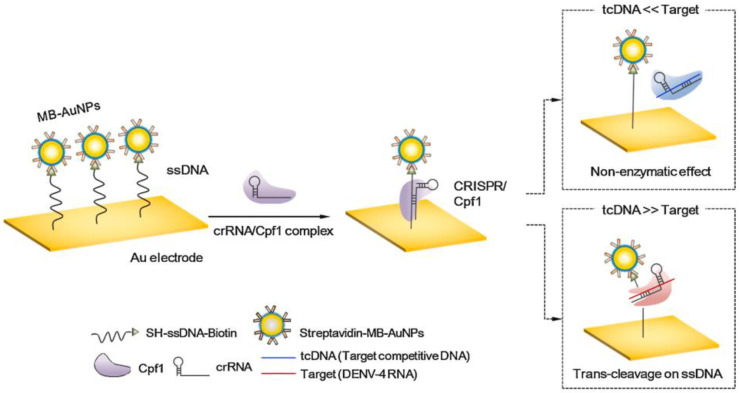
The CRISPR system for detection of RNA Dengue Virus (DENV). (Reprinted with permission from Elsevier [93]. Copyright and Licensing.).

Gootenberg et al. (2017) [89] developed a platform known as Specific High-Sensitivity Enzymatic Reporter UnLocking (SHERLOCK) which harnessed the collateral cleavage activity of Cas13a to detect specific strains of DENV and ZIKV. The platform is also capable of distinguishing pathogenic bacteria as well as identifying mutations in cell-free tumor DNA and genotype human DNA. Gootenberg et al. (2018) [90] developed a second version of SHERLOCK known as SHERLOCKv2, as a form of paper-based biosensing system for the detection of Zika virus RNA. The system is designed using guide RNA and Cas13a which recognizes the target and triggers collateral cleavage activity. The biosensor was able to achieve a detection limit as low as 20 aM. In order to address the need of the POC diagnostic platform for the detection of Ebola virus, Qin et al. (2019) [91] developed an automatic system which harnessed the collateral cleavage activity of Cas13a for degradation of target RNA. The degraded RNA fragments are measured using a custom fluorometer. The developed biosensing platform was able to achieve a result within 5 min and 20 pfu (5.45 × 10^7^ copies/mL) detection limit. The use of CRISPR/Cas12 and Cas13 for detection of diseases is illustrated in Figure 4. Figure 5 shows the CRISPR system used to detect dengue fever using Cas12a/cpf1, with a target RNA DENV. The summary of Cas systems used for the detection of tropical diseases are shown in Table 3.

## 6. Open Research Issue

The field of biosensing technology has witnessed progressive advancement in the past few years. What started merely as an electrochemical glucose biosensor has now been developed and transformed to molecular diagnosis of pathogenic disease. The main players or contributors to this transformation and innovations include the discovery of new approaches such as the CRISPR/Cas system, NA amplification techniques, nanotechnology, electronics, and material science. Despite the progress made so far in terms of the development of biosensors that function without amplifications steps, exhibition of high specificity based on SNP, and sensitivity of pM, fM, and aM concentrations, the biosensor field is still hindered by several challenges.

The current COVID-19 pandemic and the past Ebola, dengue, and Zika virus endemic have changed the landscape of clinical diagnosis from bench-lab assay to POC diagnostics. Scientists have proposed theoretical approaches and developed models and prototypes as well as a few POC devices for real-time diagnosis. Despite this progress, the developments of ideal, portable, cheap, precise, accurate, highly specific, and sensitive POC diagnostics biosensors still remain a challenge. The advancement in the field of computer science, the Internet of things (IoT), and Artificial Intelligence (AI) has opened the gateway to smart biosensors capable of collecting, storing, analyzing, and sharing data generated from biosensors in the form of numerical values or signals. However, smart biosensing technology is hampered by privacy and security issues which need to be addressed before it can be fully adopted into medical diagnosis.

## 7. Conclusions

TDs cause by DENV, ZIKV, Ebola, HIV, tuberculosis, etc., have caused havoc worldwide. Real-time, accurate, and early diagnoses of TDs is crucial for early treatment and epidemiological surveillance. Despite the wide array of clinical diagnostic approaches, including antibody, whole cell, and enzymatic-based techniques, nucleic acid-based detection approaches still remain the most sensitive and specific. The use of the RT-PCR-based approach has proved to be more efficient than antigen antibody-based methods due to its high specificity (hybridization) and amplification of target DNA.

The recent discovery of CRISPR/Cas systems in bacteria and archaea is revolutionizing the field of gene editing technology and biosensors. Several Cas systems have been identified and isolated from bacteria and programmed along with synthetic guide RNA to navigate through a long thread of genome in order to recognize a matching sequence. Scientists harness this activity in order to edit gene (insertion or deletion) for the development GMO and treatment of diseases. Several CRISPR-based biosensors have been developed including Cas9 and dCas9-based, Cas12-based, and Cas-13-based for the clinical diagnosis of bacterial and viral pathogens.

CRISPR/Cas system-based gene editing technology remains the most viable approach for eliminating inheritable diseases such as sickle cell anemia, Duchene muscular dystrophy (DMD), cystic fibrosis, Huntington’s disease, etc. The biomimetic application of the system on editing target NA has open a Pandora’s box for numerous futuristic applications on fighting diseases, enhancing features (such as designing babies who are immune to disease, increasing intelligence, enhancing eye color, etc.), and the detection of disease. Despite the hype of this technology, it is clouded by several challenges such as off-target the need for amplifications, the conversion of signals into readable output or numerical values, sensitivity beyond the femtomolar range, and ethical concerns. Thus, these challenges need to be addressed in order for this technology to reach its full potential.

## Figures and Tables

**Table 1:** Classification of CRISPR/Cas systems.

Class	Type	Adaptation	Pre-CrRNA Processing	Effector Module	Target Cleavage
Class 1	I	Cas1, Cas2, and Cas4	Cas6	Cas7 and Cas5	Cas3
	III	Cas1 and Cas2	Cas6	Cas7 and Cas5	Cas10
	IV	-	-	Cas7 and Cas5	-
Class 2	II	Cas1, Cas2, and Cas4	RNaseIII	Cas9	Cas9
	V	Cas1, Cas2, and Cas4	-	Cpf1 (Cas12) and Cas14	Cpf1 (Cas12) and Cas14
	VI			Cas13	Cas13

**Table 2:** The difference between Cas effectors.

Differences	Cas9	Cas12	Cas13	Cas14
Domains	RubC and HNH	RuvC	2 HEPN	RuvC
Target	DSDNA	SSDNA	RNA	SSDNA
Organism derived from	*Streptococcus pyogenes* *Streptococcus thermophilus* *Staphylococcus aureus*	*Prevotella* sp. *Francisella* sp. *Lachnospiraceae bacterium* ND 2006 *Acidaminococcus* sp.	*Prevotella* sp. *Leptotrichia wadei*	*Extremopile archaea*
Types of cuts	Blunt	Staggered	-	-
TracRNA	Present	Absent	Present	Present
PAM sequence	NGG	T-rich	PFS	Not required

**Table 3:** Detection of tropical diseases using the CRISPR/Cas system.

Cas System	Pathogen	References
Cas9/dCas9	*Mycobacterium tuberculosis*	[81]
	Zika and dengue virus	[82]
Cas12	Dengue virus	[94]
	*Mycobacterium tuberculosis*	[85]
	HIV	[86]
	Viral NA	[87]
Cas13	Dengue and Zika viruses	[89]
	Zika virus	[90]
	Ebola virus	[91]

## Data Availability

Not applicable.

## List of Abbreviations

**Abbreviations**	**Meaning**
A	Adenine
AchE	Acetylcholinesterase
AI	Artificial Intelligence
AIDS	Acquired Immune Deficiency Syndrome
aM	Attomolar
AuNPs	Gold Nanoparticles
Bp	Base Pair
C	Cytosine
CAS-EXARP	CRISPR/Cas9 Triggered Isothermal Exponential Amplification
CD4	Clusters of Differentiation 4
CDC	Center for Disease Control and Prevention
COVID-19	Coronavirus Disease 2019
CRISPR	Clustered Regularly Interspaced Short Palindromic Repeat
CrRNA	CRISPR RNA
CT	Computerized Tomography
CV	Cyclic Voltammetry
DNA	Deoxyribonucleic Acid
dCas9	Deactivated Cas9
DENV	Dengue Virus
DRC	Democratic Republic of Congo
DSB	Double-Strand Break
dsDNA	Double-Strand DNA
fM	Femtomolar
GMO	Genetically Modified Organism
HDR	Homologous Direct Repair
HEPN	Higher Eukaryotic and Prokaryotic Nucleotide
HOLMES	one-HOur Low-cost Multipurpose highly Efficient System
HIV	Human Immunodeficiency Virus
HR	Homologous Repair
HRP	Horseradish Peroxidase
IoT	Internet of Things
LAMP	Loop-Mediated Isothermal Amplification
LOD	Limit of Detection
KDA	Kilodalton
NA	Nucleic Acid
NASBA	Nucleic Acid Sequence-based Amplification
NASBACC	Nucleic Acid Sequence-based Amplification (NASBA)-CRISPR Cleavage
NHEJ	Non-homologous End-joining
NHPs	Non-Human Primates
NT	Nucleotide
NTDs	Non-Tropical Diseases
NTS	Non-Target Strand
MB	Methylene Blue
MTC	*Mycobacterium Tuberculosis* Complex
PAM	Protospacer Adjacent Motif
PC	Paired Cas9
PCR	Polymerase Chain Reaction
PFS	Protospacer Flanking Sequence
Pfu	Plague-forming Unit
Pm	Picomolar
POC	Point-of-Care
POCT	Point-of-Care Testing
RCA	Rolling Circle Amplification
rRT-RAA	Real-time Isothermal Reverse-transcription Recombinase-aided Amplification.
RT-PCR	Real-Time Polymerase Chain Reaction
RNA	Ribonucleic Acid
SgRNA	Single Guide RNA
SHERLOCK	Specific High-Sensitivity Enzymatic Reporter UnLocking
SSDNA	Single-Stranded DNA
SSRNA	Single-Stranded RNA
TALENS	Transcription Activator-like Effector Nucleases
TacrRNA	Transactivating CRISPR RNA
TDs	Tropical Diseases
UNICEF	United Nation International Children’s Emergency Fund
µM	Micromolar
U	Uracil
USA	United State of America
TB	Toluidine Blue
TMB	Tetramethylbenzidine
ZFN	Zinc-finger Nucleases
ZIKV	Zika Virus
